# Transcatheter tricuspid valve treatment: a growing armamentarium of devices

**DOI:** 10.1093/eurheartjsupp/suaf098

**Published:** 2025-12-26

**Authors:** Rodrigo Estevez-Loureiro, Julio Echarte-Morales, Nicole Karam, Axel Linke, Henryk Dreger, Xavier Freixa, Thomas Modine, Philip Lurz

**Affiliations:** Department of Cardiology, University Hospital Alvaro Cunqueiro, Vigo, Spain; Cardiovascular Research Group, Department of Cardiology, University Hospital Alvaro Cunqueiro, Fundación Biomédica Galicia Sur, Servizo Galego de Saude, University of Vigo, Vigo, Spain; Department of Cardiology, University Hospital Alvaro Cunqueiro, Vigo, Spain; Cardiovascular Research Group, Department of Cardiology, University Hospital Alvaro Cunqueiro, Fundación Biomédica Galicia Sur, Servizo Galego de Saude, University of Vigo, Vigo, Spain; Clemenceau Medical Center, Cardiology Department, Beirut, Lebanon; Department of Internal Medicine and Cardiology, Herzzentrum Dresden, Universitätsklinik an der Technischen Universität Dresden, Dresden, Germany; Deutsches Herzzentrum der Charité, Department of Cardiology, Angiology and Intensive Care Medicine, Campus Virchow Klinikum, Berlin, Germany; Charité—Universitätsmedizin Berlin, Corporate Member of Freie Universität Berlin and Humboldt-Universität zu Berlin, Charitéplatz 1, Berlin, Germany; Cardiology Department, Cardiovascular Institute (ICCV), Hospital Clinic, IDIBAPS, University of Barcelona, Barcelona, Spain; Department of Cardiology and Cardio-Vascular Surgery, Hôpital Cardiologique de Haut-Leveque, Bordeaux University Hospital, Bordeaux, France; Department of Cardiology, University Medical Center of the Johannes Gutenberg, Mainz, Germany

**Keywords:** Tricuspid regurgitation, Transcatheter tricuspid valve interventions, Tricuspid transcatheter edge-to-edge repair, Transcatheter tricuspid valve replacement, Spacer, Annuloplasty

## Abstract

Tricuspid regurgitation is a frequent and undertreated condition associated with poor outcomes. Over the past decade, transcatheter tricuspid valve interventions have emerged as a valuable alternative for high-risk patients. Edge-to-edge repair is the most widely adopted approach, while replacement technologies are advancing rapidly and offer the potential for near-complete elimination of regurgitation. In parallel, annuloplasty, spacers, and caval devices are broadening the therapeutic spectrum across diverse anatomies. This review outlines the expanding device armamentarium and the key opportunities and challenges in this evolving field.

## Graphical Abstract

**Graphical Abstract:**
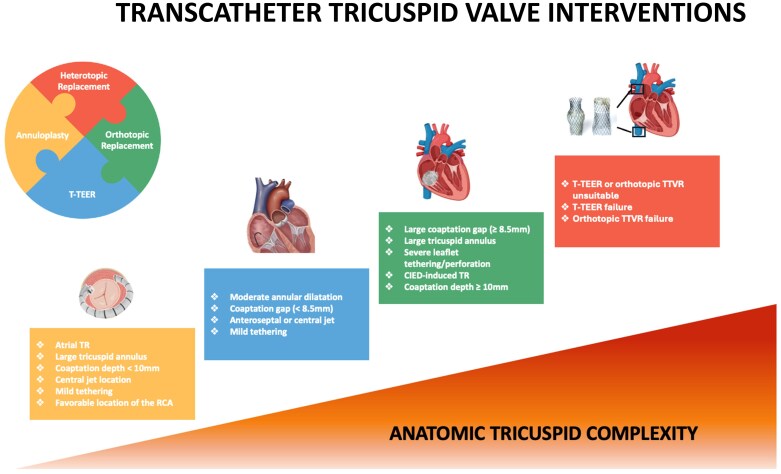
**Tailored approach to device selection in TTVI.** CIED, cardiac implantable electronic device; RCA, right coronary artery; TR, tricuspid regurgitation; T-TEER, transcatheter tricuspid edge-to-edge-repair; TTVR, transcatheter tricuspid valve replacement; TTVI, transcatheter tricuspid valve interventions

## Introduction

Tricuspid regurgitation (TR) is a common valvular disorder associated with significant morbidity and mortality. In the USA, moderate-to-severe TR affects more than 1.6 million individuals,^1^ and its prevalence rises sharply with age, reaching nearly 4% in those over 75 years.^2^ Surgical treatment of isolated TR has traditionally been associated with high risk, with operative mortality rates close to 10% in some reports, particularly following valve replacement as opposed to repair.^3,4^ Such unfavourable outcomes are often the consequence of late referral, when right-sided cardiac and end-organ damage are already advanced, and are further aggravated by the advanced age and comorbidities that characterize this patient population.

**Figure 1:**
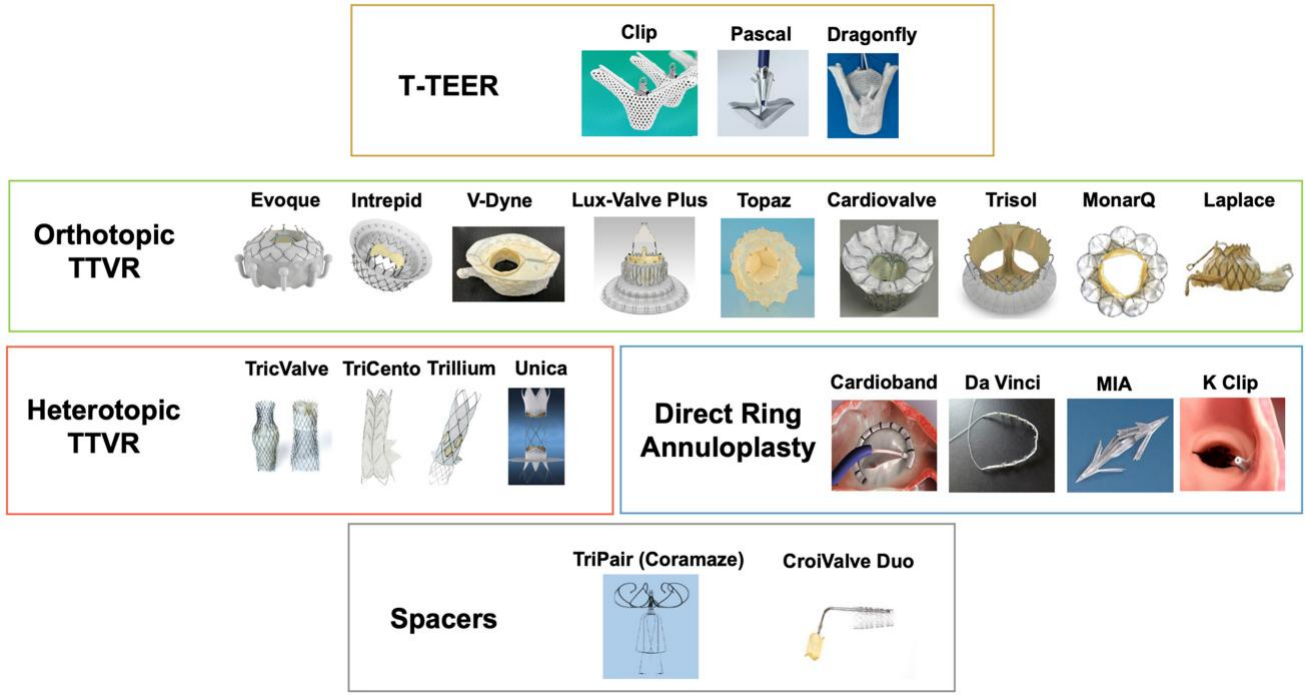
Transcatheter tricuspid landscape. T-TEER, tricuspid transcatheter edge-to-edge repair. TTVR, transcatheter tricuspid valve replacement.

Against this background, transcatheter therapies have been developed to address an important unmet clinical need. Among them, transcatheter edge-to-edge repair (TEER) is currently the most widely used approach, supported by an expanding body of clinical evidence.^5,6^ Nevertheless, its effectiveness may be limited in anatomically complex scenarios, such as large coaptation gaps, device-related TR, or severe leaflet tethering. These limitations have driven the development of other approaches, such as annuloplasty devices, transcatheter tricuspid valve replacement (TTVR), and spacer-based systems.^7-9^ Together, these innovations are expanding the interventional toolbox and are progressively reshaping the therapeutic landscape of TR (*Figure 1*).

This review provides an overview of the current device-based strategies for transcatheter treatment of TR, their mechanisms of action, available clinical evidence, and emerging role in clinical practice (*Graphical Abstract*).

## Transcatheter edge-to-edge repair

TEER addresses atrioventricular regurgitation by approximating opposing leaflets at the origin of the regurgitant jet. This method first appeared in mitral regurgitation (MR) and became worldwide adopted following initial experiences in primary MR.^10,11^ It is now widely used to treat both primary and secondary MR. Although TEER has been effective in MR repair, it took almost a decade until the initial usage in TR repair, owing to the complexity of tricuspid valve anatomy, the challenge of visualizing the leaflets with transoesophageal echocardiography, and design limitations of earlier devices developed for the mitral position.^12^

Two dedicated TEER systems are approved for the European market to date: the TriClip® (Abbott, April 2020) and PASCAL® (Edwards Lifesciences, May 2020) devices. Of these, only the TriClip received FDA approval, granted in April 2024, whereas comparable systems have been developed and introduced in Asia.^13^

TEER has been rapidly adopted as the standard transcatheter approach for TR in many centres due to its favourable safety profile, encouraging early results, and the vast experience and familiarity of many operators with mitral TEER procedures. Although post-procedural TR ≥ moderate has been reported in about 10–20% of patients in both randomized trials (with highly selected populations) and real-world registries (with broader inclusion criteria), its main advantage compared to TTVR is an excellent safety profile and very low in-hospital mortality.^14,15^

TTVR can effectively eliminate TR, but it also comes with higher periprocedural risks, such as greater in-hospital mortality, conduction problems that may require a permanent pacemaker, and more bleeding complications.^8,16^ These issues are in part driven by the need for stricter anticoagulation to prevent valve thrombosis and hypo-attenuated leaflet thickening (HALT). *Table 1* summarizes the key performance metrics of contemporary tricuspid devices, and *Table 2* highlights the clinical relevance of different risk score tools for patients with severe TR.

**Table 1:** Overview of the most commonly used TTVI devices

Device	CE mark	FDA approval	Study	Procedural success %	Renal replacement therapy %	Severe bleeding %	Major access site complications %	PPM %	TR severity at 1 year %	1-year Mortality %
TriClip	Yes (2020)	Yes (2024)	TRILUMINATE Pivotal Trial^14^	NA	NA	5.2	1.7	2.9	TR ≤ mild NA TR ≤ moderate 88.1	8.8
PASCAL			CLASP TR^17^	87.5	0.0	9.2	3.1	NA	TR ≤ mild 44.0 TR ≤ moderate 86.0	10.8
	Yes (2020)	Yes (2022)	TriCLASP^18^	85.7	2.3	5.8	0.0	NA	TR ≤ mild 55.7 TR ≤ moderate 87.3	11.0
Evoque			TRISCEND^19^	93.0	3.4	25.5	4.0	13.3	TR ≤ mild 97.6 TR ≤ moderate 100.0	9.1
	Yes (2023)	Yes (2024)	TRISCEND II^8^	NA	3.1	15.4	3.1	27.8	TR ≤ mild 95.3 TR ≤ moderate 99.1	NA
Lux-Valve Plus			TRAVEL II^20^	95.6	1.0	0.0	NA	2.1	TR ≤ mild 95.3 TR ≤ moderate 98.9	4.2
	No	No	TRINITY^21^	92.0	0.0	4.0	0.0	11.9	NA	NA
Cardiovalve	No	No	Compassionate use^22^	90.0	NA	20.0	NA	10.0	NA	NA
TricValve	Yes (2021)	No	TRICUS study^23^	94.0	NA	17.1	NA	3.0	NA	NA
Cardioband			Early feasibility study^24^	82.6	0.0	35.1	8.1	NA	TR ≤ mild 23 TR ≤ moderate 73.0	13.5
	Yes (2018)	No	TriBAND^25^	83.9	3.3	11.5	6.6	1.6	NA	NA

CE, Conformité Européenne; FDA, US food and drug administration; NA, not available; PPM, permanent pacemaker; TR, tricuspid regurgitation; TTVI, transcatheter tricuspid valve interventions.

**Table 2:** Prognostic risk scores developed for patients with significant tricuspid regurgitation

Score (year)	Population	Endpoint	Variables included	Range/risk strata
LaPar Score^26^ (2018)	Patients undergoing isolated tricuspid valve surgery *n* = 2050	Operative mortality and major morbidity	Age; sex; prior stroke; haemodialysis; EF <55%; chronic lung disease; advanced NYHA class; reoperation; urgent/emergency status	0–10 + points; predicted mortality 2% to 34% across score values; predicted morbidity 13% to 71% across score values
Tri-Score^27^ (2022)	Patients undergoing isolated tricuspid valve surgery *n* = 466	In-hospital mortality	Age ≥70; NYHA III–IV; right-heart failure signs; furosemide ≥125 mg/day; eGFR <30 mL/min; elevated total bilirubin; LVEF <60%; moderate/severe RV dysfunction	0–12 points (low <4; intermediate 4–5; high ≥6); predicted mortality increased from 1% to 65% as scores rose from 0 to ≥9
TRIO Score^28^ (2022)	Patients with ≥ moderate TR *n* = 13 608	All-cause mortality over 10 years	Age; male sex; creatinine ≥2 mg/dL; heart failure; chronic lung disease; AST ≥40 U/L; heart rate ≥90; severe TR	0–12 points (low <4/intermediate 4–6/high risk ≥7); high risk was associated with a 10-year mortality of 79%
Hochstadt Score^29^ (2022)	Conservatively managed significant TR *n* = 1701	1-year mortality	Age; BMI ≤25; liver disease; chronic lung disease; eGFR; Hb; LVEF ≤30%; RV dysfunction; RAP; stroke volume index ≤30; LVEDD ≤45 mm	0–17 points; predicted mortality increased from 0% to 100% as scores rose from 0 to ≥16
Wang Score^30^ (2022)	Isolated ≥ moderate TR *n* = 9045	1-year mortality	Age; MI; PAD; chronic lung disease; creatinine >1.4 mg/dL; loop diuretic use; Hb <10 g/dL; platelets <15 K/μL; INR >1.5; albumin <3 g/dL; RV dysfunction; RVSP >50 mmHg	0–16 points; predicted mortality 2.3% to 67% across score values
Trivalve Score^31^ (2024)	Patients with severe TR undergoing TTVI	1-year mortality or HF hospitalization	Atrial fibrillation; eGFR <30 mL/min; elevated liver enzymes (GGT/bilirubin); right-sided heart failure signs; LVEF <50%	0–4.5 points; optimal cut-off ≥2.5 for the primary endpoint

AST, aspartate aminotransferase; BMI, body mass index; EF, ejection fraction; eGFR, estimated glomerular filtration rate; GGT, gamma-glutamyl transferase; Hb, haemoglobin; HF, heart failure; INR, international normalized ratio; LVEF, left ventricular ejection fraction; LVEDD, left ventricular end-diastolic diameter; MI, myocardial infarction; mmHg, millimetres of mercury; NYHA, New York Heart Association; PAD, peripheral artery disease; RAP, right atrial pressure; RV, right ventricle; RVSP, right ventricular systolic pressure; TR, tricuspid regurgitation; TTVI, transcatheter tricuspid valve intervention.

## The TriClip transcatheter valve repair system

TriClip has the specificity to feature a delivery system designed specifically to navigate the anatomy of the right heart. The system allows for precise steering, positioning, and leaflet grasping under echocardiographic and fluoroscopic guidance. Multiple clip sizes are available, similar to the mitral valve, though the XTW is the most widely used, justified by its potential to achieve a better reduction in TR. This is due to the larger surface of coaptation, resulting from broader and larger arms that allow for a deeper leaflet insertion.^5,14^

The US FDA evaluated the TriClip system for regulatory approval through the TRILUMINATE Trial, which was a prospective, single-arm, non-randomized study.^32,33^ The trial included 85 patients with severe TR symptoms who were deemed unfit for surgery and treated with TriClip-assisted tricuspid TEER. The study's primary focus was on evaluating safety and device performance metrics, including major adverse event rates (MAE), procedural success, and echocardiographic improvement in TR severity, to determine the effectiveness of the TriClip system. Ninety-four percent of patients were still free of MAE at 6 months, and in all patients, devices were successfully implanted. After a year, scores on the New York Heart Association (NYHA) class and the Kansas City Cardiomyopathy Questionnaire (KCCQ) showed significant improvements, indicating enhanced patient quality of life. Additionally, 87% of patients experienced a decrease in TR severity by at least one grade.

Subsequently, the TRILUMINATE Pivotal Randomized Controlled Trial, a multicenter, prospective, randomized controlled trial, was conducted to evaluate the efficacy of TriClip in conjunction with optimal medical therapy (OMT) vs. OMT alone.^14^ Enrolling 350 patients with severe, symptomatic TR, the study randomized participants in a 1:1 fashion, with 175 patients receiving TriClip and 175 continuing on medical therapy alone. The primary endpoint was a hierarchical composite of all-cause mortality, tricuspid valve surgery, heart failure hospitalization, or a clinically significant (≥15 point) improvement in KCCQ score at 12 months, analysed with a win-ratio approach. Patients treated with TriClip had significant improvements in quality of life, NYHA functional class, and TR reduction compared to those on medical therapy alone. In particular, 50% of patients in the device group experienced a ≥15-point increase in KCCQ score, compared to 26% in the OMT group. However, no differences were observed in mortality of HF hospitalization rates at 1-year follow-up. At 24 months, patients who underwent T-TEER with TriClip continued to demonstrate a meaningful and durable reduction in TR severity, with over 80% maintaining TR at moderate or less.^6^ Improvements in functional status were also preserved, with the majority of treated patients remaining in NYHA class I or II. KCCQ remained significantly improved, with an average increase of over 15 points sustained from baseline. Importantly, a notable number of patients initially randomized to OMT alone crossed over to receive TriClip during the follow-up period due to symptom progression. These crossover patients exhibited similar improvements in TR severity, functional class, and quality of life after device implantation, further validating the efficacy of TriClip in this population. Importantly, hospitalizations for heart failure were consistently lower in the TriClip group compared with the medical therapy arm over 2 years, while mortality rates remained similar between groups.^6^

The TRI.fr randomized clinical trial, funded by the French Ministry of Health, compared T-TEER using TriClip plus OMT against OMT alone.^34^ In this trial, 300 patients with symptomatic severe TR (mean age≈79 years, 64 % female) were randomized to T-TEER + OMT (*n* = 152) or OMT alone (*n* = 148), with one-year follow-up. Overall, 74 % of T-TEER + OMT patients achieved improvement on a composite clinical outcome, which included NYHA class, patient global assessment, and KCCQ, compared to 40.6 % with OMT alone (*P* < 0.0001). At 1 year, 21.8 % of T-TEER + OMT patients still had TR worse than moderate, vs. 89 % in the OMT group (*P* < 0.001). The average change in KCCQ score was +15.9 vs. + 0.4. The composite endpoint—including death, surgery, HF hospitalization, or ≥15-point KCCQ improvement—showed a hazard ratio of 2.06 (95 %CI 1.38–3.08; *P* = 0.0004) in favour of T-TEER. Importantly, MAEs were similar between arms at approximately 16–19 %. Although TRI.fr did not show a significant difference in hard outcomes like mortality or hospitalization alone, it reinforced TriClip’s value in improving symptoms and quality of life.

Real-world data further validate these trial findings. The bRIGHT study is a prospective, multicentre, post-market evaluation of TriClip in 511 patients across 26 European centres.^5^ The study confirmed the safety and effectiveness of TriClip outside of controlled trial settings. At 30 days, 77% of patients had TR moderate or less, and 79% of patients improved to NYHA class I or II, a marked improvement from the 20% baseline. Moreover, over half of the patients had a KCCQ improvement of at least 15 points, indicating a significant enhancement in day-to-day functioning. The rate of MAE was low, at just 2.5%, reinforcing the device's excellent safety profile in broader clinical practice.

## The PASCAL transcatheter valve repair system

The PASCAL device (Edwards Lifesciences) is a TEER system, which has been used in both the mitral and tricuspid positions.^35^ It is part of the broader category of transcatheter valve therapies aimed at reducing the severity of regurgitation without the need for open-heart surgery. It consists of a unique central spacer attached to two paddles and clasps. The distinctive component of the central spacer that fills the regurgitant orifice, is believed to further enhance the reduction of TR, but head-to-head studies comparing different TEER technologies are still missing.^36^ Using transoesophageal echo guidance, the opened PASCAL device is usually pulled from the right ventricular cavity to the atrioventricular-junction to grasp the leaflets on the paddles, which are then captured by lowering the clasps that can be simultaneously or independently controlled. The PASCAL Ace device carries a narrower and shorter profile. Both implants (PASCAL and PASCAL ACE) can be elongated and narrowed, which is useful in case subvalvular chordal entanglement potentially occurring during device manipulation. The design of the flexible, steerable delivery catheter in conjunction with the delivery sheath facilitates accurate placement within the complex anatomy of the tricuspid valve. The device performance was evaluated in registries and single-arm studies, but data from randomized clinical trials using the PASCAL in TR are still missing. CLASP TR EFS was a single-arm, multicentre, prospective early feasibility study that used the PASCAL transcatheter valve repair system for the treatment of TR in the USA.^37^ Patients with symptomatic TR despite OMT were eligible for the study after review by the heart team. Feasibility endpoints included safety (composite MAE rate), echocardiographic, clinical, and functional endpoints. The study included 34 patients with a mean age of 76 years (53% female), with a mean Society of Thoracic Surgeons score of 7.3%; 97% had severe or greater TR, and the majority was in NYHA functional class III/IV (79%). Twenty-nine patients received implants and the MAE was 5.9%; however, none of the patients experienced cardiovascular mortality, stroke, myocardial infarction, renal complication, or reintervention early after treatment. At 30 days, 85% of the 29 patients achieved a TR severity reduction of at least 1 grade, with 52% with moderate or less TR (*P* < 0.001) and 89% of the patients improved to NYHA functional class I/II (*P* < 0.001), which was accompanied by an improvement in mean 6-min walk distance by 71 metres (*P* < 0.001), and in mean KCCQ score by 15 points (*P* < 0.001). These results were in line with data from TriCLASP, which was a prospective, single-arm, multicenter European post-market clinical follow-up study to evaluate the safety and performance of the PASCAL system in patients with severe or greater TR.^38^ At 30 days, TR was significantly reduced, and clinical, functional, and quality of life outcomes significantly improved in a cohort of 74 patients with a mean age of 80.3 years in an almost similar manner as in CLASP TR.

The positive early results of the two above-mentioned trials were confirmed by the 12-month data from CLASP TR that contained data from 65 patients (mean age: 77.4 years; 55.4% female; 97.0% with severe to torrential TR).^17^ At 30 days, in this cohort, cardiovascular mortality and stroke rate were 3.1% and 1.5%, respectively, and no device-related reinterventions were reported. Between 30 days and 1 year, three additional cardiovascular deaths (4.8%), two strokes (3.2%), and one unplanned or emergency reintervention (1.6%) occurred. At 1-year post procedure, 31 of 36 (86.0%) patients had moderate or less TR, which was accompanied by improvement in NYHA functional class, 6-minute walk distance increased by 94 metres, KCCQ scores improved by 18 points (*P* <0.001) and freedom from all-cause mortality and heart failure hospitalization were 87.9% and 78.5%, respectively.

The largest piece of evidence regarding the use of PASCAL for treatment of TR is reported in the PASTE (PASCAL for Tricuspid Regurgitation-a European registry) study, which is an investigator-initiated, multicentre, retrospective, and prospective observational cohort analysis conducted across 16 European heart valve centres including consecutive patients treated with the PASCAL transcatheter valve repair system from February 2019 to November 2023.^39^ The study included 1059 high-risk patients (mean age 79 ± 9 years; 53% female; TRI-SCORE risk 23% ± 18%; 87% NYHA functional class III/IV). At least severe TR was present in 96% of patients. Intraprocedural success was achieved in 85%, and TR reduced to less than moderate in 87%. Independent predictors for a post-procedure residual TR of more than moderate were coaptation gaps ≥8 mm, tenting height ≥10 mm, the presence of a transvalvular lead, right ventricular dilatation >42 mm, and massive/torrential TR at baseline. At 1 year, 83% of patients showed ≤ moderate TR and 66% were in NYHA class I/II (vs. 17% at baseline; *P* < 0.001). Improvement in the delivery system (early generation vs. precision) resulted in a greater TR reduction to trace/mild, shorter procedure times by approximately 30 min and slightly higher but significant clinical success rates according to the Tricuspid Valve Academic Research Consortium at 30 days and 1 year of 56% vs. 50%, respectively. Higher centre experience (≥21 patients/year) was associated with better intraprocedural and clinical success.

The results of the above-mentioned studies reinforce the potential of the PASCAL system as a safe and effective minimally invasive option for patients with severe TR who are high-risk surgical candidates. The trial's findings support the growing role of transcatheter approaches in managing complex valvular diseases, offering symptom relief and quality of life improvements. Continued follow-up and larger-scale studies are anticipated to establish the long-term durability of the device and its impact on clinical outcomes such as hospitalization and survival. As the evidence base expands, TEER may become an integral part of the treatment algorithm for TR.

## Orthotopic transcatheter tricuspid valve replacement: current device portfolio and unmet challenges

Technical and clinical assessment and initial commercial application are focused on several transcatheter TTVR system alternatives designed to alleviate anatomical and functional problems posed by the individual application of the tricuspid valve. Efficient screening and procedural planning require an in-depth understanding of complex tricuspid valve anatomy.^40^ Multimodality imaging is necessary given that transoesophageal echocardiography may be attenuated by acoustic shadowing in an appreciable minority of cases, particularly in patients with a history of cardiac intervention or device implantation.^41^ Cardiac computed tomography (CT) is critical in annular measurement, right heart, and venous anatomy to guide device and access strategy. The non-calcified dynamic annulus of the tricuspid valve, thin right ventricular myocardium, and close vicinity to the conduction system make device anchoring difficult and predispose to conduction disturbances, even permanent pacemaker implantation.^9,42^ Vascular access is typically transfemoral but sometimes transjugular and requires individualization.

**Figure 2:**
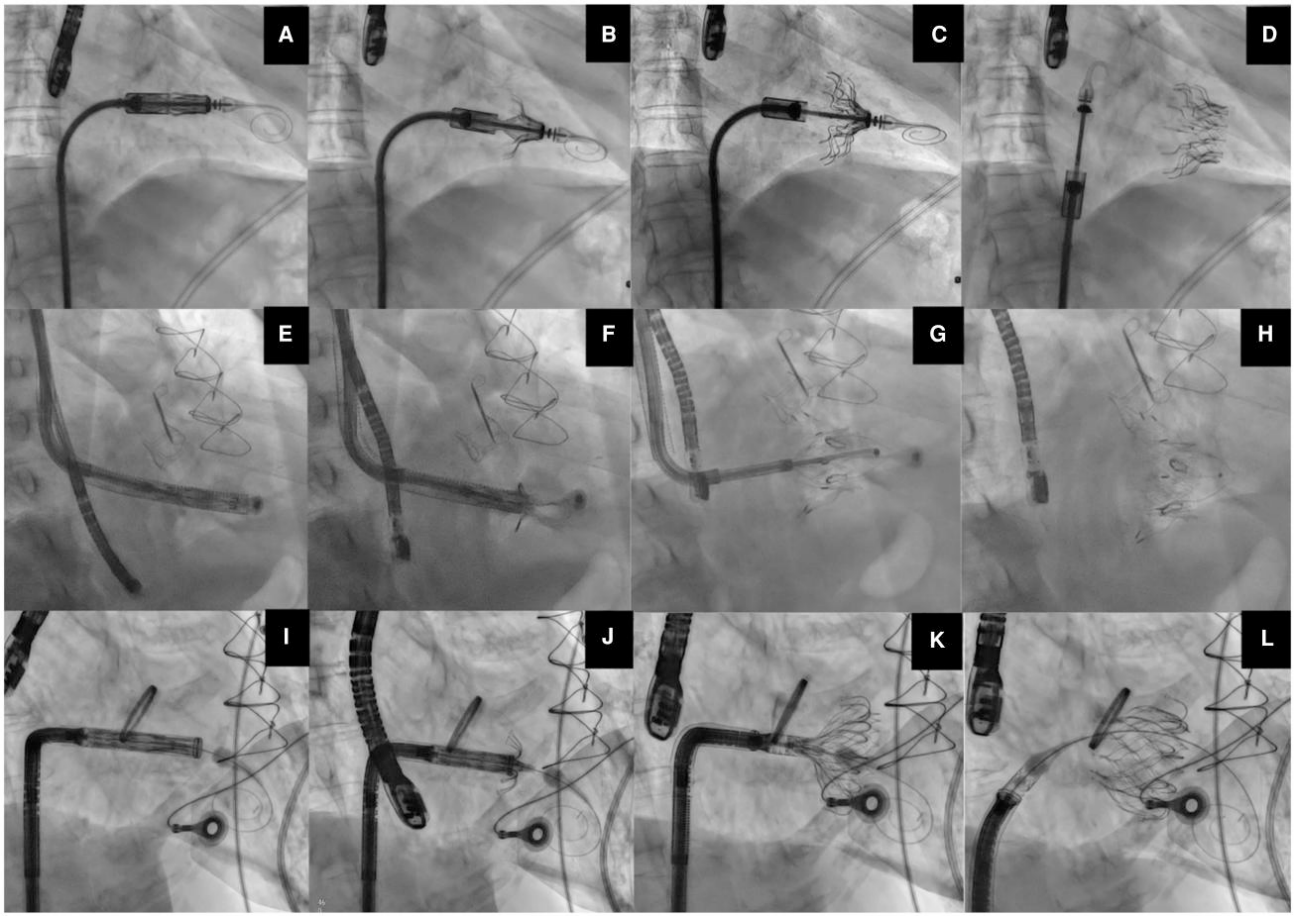
Intra-procedural fluoroscopic image of 3 percutaneous valve replacement systems. A–D: Cardiovalve; E–H: Lux-Valve; I–L: Evoque.

Clinical challenges may include the risk of afterload mismatch, which may lead to acute right ventricular failure following abrupt elimination of TR, particularly in patients with pre-existing right ventricular dysfunction. Importantly, this is rare in patients undergoing T-TEER,^43^ and for TTVR the data are scarce, with reported rates ranging from 0% to 2%. To mitigate this risk, pre-procedural optimization of volume status and right ventricular function is crucial. Furthermore, while post-TTVR anticoagulation strategies are essential, they remain unstandardized, despite the high incidence of atrial fibrillation and the associated thrombosis risk. Likewise, severe bleeding complications have also been reported peri-procedurally.^8,19^ Additionally, the long-term durability of the device remains uncertain due to the novelty of TTVR, although early outcomes are promising. Finally, the long-term effects of residual or recurrent TR on survival and heart failure are yet to be fully understood. *Figure 2* shows the fluoroscopic implantation steps of three TTVR devices used in the contemporary era.

### EVOQUE

The EVOQUE valve (Edwards Lifesciences) is a self-expanding nitinol frame valve with bovine pericardial leaflets, delivered via a 28Fr transfemoral catheter. It anchors using nine subvalvular arms positioned under leaflet hinges, followed by expansion of an inner frame. This system has the most extensive clinical experience among TTVR devices and was the first to receive CE mark and FDA approval. The TRISCEND II trial randomized 400 patients with severe TR to receive either TTVR plus OMT or OMT alone.^8^ The primary hierarchical endpoint used the win ratio, encompassing mortality, right ventricular assist device implantation or transplant, reintervention, heart failure hospitalization, and improvements in quality of life as evaluated by the KCCQ, NYHA class, and 6-min walk test. At 1 year, TTVR demonstrated a win ratio of 2.02 favouring the intervention group. Pairwise analyses showed greater benefits with TTVR in mortality (14.8% vs. 12.5%), reintervention (3.2% vs. 0.6%), KCCQ-OS improvement (23.1% vs. 6.0%), NYHA class improvement (10.2% vs. 0.8%), and 6-min walk test (1.1% vs. 0.9%). When evaluated separately, all-cause death was similar among the groups (10% vs. 9.7%). Major bleeding (15.4% vs. 5.3%) and permanent pacemaker implantation (17.4% vs. 2.3%) were more frequent with TTVR. On the other hand, in a real-world cohort of 176 patients, significant clinical improvements were noted at 30 days: 98.4% had TR reduced to mild or none, and NYHA class I/II increased from 20.2% to 79.7%. Hepatorenal function improved significantly. Patients with massive/torrential TR improved more functionally, whereas those with severe right ventricular dysfunction had a higher failure risk.^16^ This aligns with a recent TRISCEND II sub-analysis based on baseline TR severity, in which patients with massive or torrential TR, compared with controls, had lower rates of HF hospitalization and of the composite of all-cause mortality and first HF hospitalization.^44^

### LuX-Valve Plus

The LuX-Valve Plus (Ningbo Jenscare Biotechnology Co., Ningbo, China) is a trileaflet bovine pericardium valve with two lateral subvalvular anchors and a ventricular anchor that is fixed to the interventricular septum. It is delivered through the jugular vein using a 31Fr steerable system. The Lux-Valve has a self-adaptative fabric skirt and anchors itself by apposition to the base of the right atrium rather than through radial forces. The ventricular septum anchor contains a three-pronged curved anchoring needle that is inserted into the interventricular septum to prevent valve migration. At 1-month follow-up, the TRAVEL II trial, investigating 96 patients undergoing transjugular implantation of the LuX-Valve system, reached a 97% device success.^45^ The 30-day composite occurrence of adverse events (death, myocardial infarction, stroke, severe paravalvular leak, and new-onset atrioventricular block necessitating pacemaker implantation) occurred in 6.5% of patients. The 30-day outcomes of the TRINITY Trial reached a TR reduction to ≤2 + in 95.7% of patients, with a composite event rate of 14.8%, of which 4% had severe bleeding and 8.7% of patients had permanent pacemaker implantation due to atrioventricular block. At 30 days, the full analysis set (FAS) and roll-in groups demonstrated significant reductions in TR grade, significant reduction in NYHA functional class, and significant improvement in quality of life, with 95.7% (FAS) and 91.7% (roll-in) having no greater-than-moderate TR and a mean increase in the KCCQ score of approximately 14 and 18 points, respectively.^21^

### Intrepid

The Intrepid TTVR system (Medtronic, MN, USA) features a dual-stent bovine pericardial valve designed for transcatheter implantation via a 35Fr venous delivery system, with a smaller 29Fr system under development. It comes in two outer frame sizes (44 and 48 mm), both with a 27 mm inner stent frame. The outer frame has been modified compared to the mitral device to better accommodate tricuspid anatomy. What distinguishes the Intrepid valve is its direct annular anchoring mechanism that relies on radial force. This technology reduces the need for leaflet capture or anchoring and thus allows easier imaging during the procedure. An early feasibility trial of the Intrepid TTVR system is currently recruiting patients (NCT04433065) to evaluate implant-related serious adverse events at 30 days as the primary endpoint. The secondary outcomes are the change in grade of TR, procedural success, stenosis of the tricuspid valve, and the improvement in functional class in the NYHA.

### Cardiovalve

The Cardiovalve (Cardiovalve Ltd., Or Yehuda, Israel) features a self-expanding nitinol frame that anchors to the native leaflets. Its inner frame contains three bovine pericardial leaflets. The nitinol frame provides 24 grasping points designed for atraumatic anchoring and secure fixation to the native valve. The delivery system utilizes a 32Fr capsule with a 24Fr shaft. Results with the cardiovalve system were reported in 20 patients receiving compassionate use for severe functional TR. Device success was achieved in 90% of cases. At 30 days, TR was reduced to ≤1 + in 95% of patients; the mortality rate was 10%, and HF hospitalization within the first month after TTVR occurred in 4.2% of the cohort.^22^ The TARGET study is a multicenter, prospective, single-arm study designed to evaluate the safety and performance of the cardiovalve TTVR (NCT05486832). The primary outcomes are freedom from device or procedure-related adverse events and reduction in TR grade assessed at 30 days.

### Trisol

The Trisol valve (Trisol Medical, Yokneam, Israel) consists of a self-expanding conical nitinol stent that supports a single bovine pericardial dome-shaped leaflet attached at two opposing central commissures, which functions as a bileaflet valve. Its design aims to achieve a high closing volume, thereby reducing the immediate increase in afterload associated with complete TR elimination. Anchoring of the valve is achieved through axial force between the ventricular porcine pericardium and atrial polyester skirts. The valve offers both retrievability and repositionability, and access is obtained using a 30Fr transjugular delivery system. An early feasibility study with trisol is currently underway in the USA (NCT04905017). The primary endpoint includes the rate of device-related serious adverse events, technical success, procedural success, and change in TR from baseline.

### Topaz

The Topaz TTVR system (TRiCares, Munich, Germany) has a two-frame design with an inner and outer stent, like other devices. The soft outer stent is designed to align smoothly with the patient's native annulus, securely anchoring the prosthesis within the tricuspid valve while confirming the shape and movement of the right ventricle. The inner stent supports the three-leaflet valve independent of external stent deformation, maintaining its circular shape even with significant motion of the outer stent. This atraumatic anchoring system intends to ensure a secure fit with a low risk of harming native structures. The system is delivered via the femoral vein through a 29Fr steerable introducer. The Tricares Topaz Transfemoral Tricuspid Heart Valve Replacement System First in Human Trial (TRICURE) is currently recruiting patients to measure a hierarchical composite endpoint including all-cause mortality, re-hospitalization for heart failure, re-intervention for failed tricuspid intervention, and KCCQ worsening at 30 days (NCT05126030).

### Vdyne

The Vdyne valve (Vdyne, Maple Grove, Minnesota) is available in five external frame sizes (140–180 mm perimeter) with the same 30 mm inner valve diameter to fit a large range of annular sizes. It is made of porcine pericardium and is implanted through transfemoral access using an innovative side-loaded delivery system. It incorporates a pop-off system to accommodate any afterload mismatch that may form after implantation. At present, one clinical trial is evaluating the safety and performance of Vdyne transcatheter tricuspid valve replacement (NCT05797519). The planned enrolment is 40 patients, and one of the primary outcomes is the percentage of subjects with device and/or procedure-related MAE within 30 days of the procedure.

### MonarQ

The MonarQ valve (InQB8 Medical Technologies) implant comprises two nitinol frames (an atrial frame and a ventricular frame with nine ventricular arms), polyester sealing skirts, and a central 30 mm-diameter trileaflet bovine bioprosthetic valve. The anchoring mechanism relies on leaflet grasping. The delivery system is designed for both transfemoral and transjugular access, and the current system is available in two sizes (medium and large) to treat patients with an annulus size ranging from 45 to 60 mm. Early compassionate use of the MonarQ TTVR demonstrated a favourable safety profile, high technical success, significant TR reduction, and meaningful clinical improvement. An early feasibility study is currently ongoing (NCT04433065), with an estimated enrolment of 150 patients and a primary endpoint of implant or delivery-related serious adverse event rate.

### Laplace

The Laplace system consists of a self-expanding nitinol frame carrying a trileaflet valve made from porcine pericardium. Its design combines a posterior flap with two anchors that press against the posterior annulus, along with an anterior flap that engages the right ventricular outflow tract to provide stability. The device is delivered through the jugular vein using a 26Fr system, which allows controlled, coaxial deployment with the option to reposition, and it can accommodate very large annuli with perimeters up to 177 mm.

So far, 35 patients have been treated in the early feasibility study (EFS). No mortality has been reported, two patients (5.7%) experienced major access-site complications, two patients(5.7%) underwent unplanned tricuspid valve-reintervention, and three patients (8.5%) required pacemaker implantation due to conduction disturbances. The results show consistent technical success with effective reduction of TR associated with KCCQ and NYHA functional class improvement.^46^

## Heterotopic transcatheter tricuspid valve replacement

Edge-to-edge repair, annuloplasty, and orthotopic replacement are best performed with appropriate anatomy and ideal imaging but are often ineligible in patients with complex anatomy or technical challenges.^47^ Heterotopic caval valve implantation (CAVI), or stent-based bi-caval stenting with valve implantation, is designed to alleviate systemic venous congestion from severe TR by reducing symptoms and venous pressures and backflow.^48^ The method has the advantage of a streamlined fluoroscopic procedural workflow by eliminating navigation and guidance around the tricuspid valve and by making it an option in patients deferrable from TEER or orthotopic TTVR. However, non-dedicated bi-caval devices have important limitations of difficulty in an anatomic fit and anchoring and in preventing embolization. Thus, reproducible optimum results have been elusive.^49^

### TricValve

The TricValve® system (Products & Features GmbH, Vienna, Austria) is a dedicated CAVI device composed of two self-expanding nitinol stents with bovine pericardial leaflets, designed for deployment in the superior vena cava (SVC) and inferior vena cava (IVC). By implanting valves in these large veins, the system decreases venous pressure, reducing peripheral oedema and hepatic and renal congestion caused by severe TR. The TricValve® system helps manage the regurgitant volume in the right atrium, and if the right ventricle preserves function and pulmonary pressures are moderate, cardiac output might be enhanced by an increased preload.^50^

The single-arm TRICUS Euro study showed strong procedural success, low peri-procedural complications, and marked functional status and quality of life improvement at 6 months in 35 high-surgical-risk patients with severe TR. Symptom improvement and diuretic reduction were also observed.^51^ Longer follow-up and compassionate use reports have supported these encouraging results with beneficial effects on right ventricular size.^52^

However, several uncertainties remain regarding the long-term impact of CAVI therapy, including the behaviour of the right atrium, right ventricular function in the presence of persistent TR, and risks of prosthesis degeneration or thrombosis.^53^ Additionally, the implantation of the TricValve® system's two separate devices poses challenges related to anatomical variability and potential valve migration, which can affect the system's effectiveness.^54^ To address these limitations, novel approaches involving cross-caval stents that host valves to prevent tricuspid backflow have been developed.

### Tricento

Tricento (NVT AG, Muri, Switzerland) is a custom-made stent graft extending from the IVC to the SVC, featuring a lateral bicuspid valve permitting flow into the right atrium. Recent experience in 21 patients demonstrated significant functional improvement and low procedural adverse events.^55^ The device was associated with a significant functional improvement and with low procedural adverse events. Although initial results have been promising, the device is custom-made and presents greater exclusion features than TricValve, which makes it less generalizable. Likewise, the development of cases of stent fractures has led to the redesign of the stent frame.

### Trillium and Unica

Two additional cross-caval devices, Trillium™ and Unica™ (Innoventric, Israel), offer alternative solutions. Trillium™ consists of a fabric-covered stent with radiopaque markers for precise fluoroscopic deployment. It has three lateral valves that can be individually crossed without affecting the others’ function. A sealing skirt prevents leaks into hepatic circulation while avoiding suprahepatic vein obstruction, a key limitation of other heterotopic valves.^56^

Unica™ features a single nitinol stent extending from SVC to IVC, hosting two trileaflet bovine pericardium bioprosthetic valves covered by PET fabric.^57^ The valves at the SVC and IVC levels are covered by PET fabric, and the system also comes with a 70 mm PET skirt at the IVC level to seal at the floor of the right atrium and prevent IVC backflow. Different from Trillium, the central portion of the stent is bare metal, allowing future access to the right atrium and ventricle. Lurz et al. reported a multicenter registry including 20 patients. Device implantation was successful in all cases, with no intraprocedural deaths or conversion to surgery. TR severity at the level of the Trillium device was reduced in all cases, and this was associated with an improvement in functional class.^58^ Both devices are promising in the field and present the clear advantages of only fluoro-guided fast deployment and prevention of hepatic back-flow by the skirt.

### Spacers

They achieve central coaptation of the leaflet and central reduction in regurgitant flow with the aid of a central spacer. The spacer is linked to an anchor, usually either a caval stent or an implant anchored in the heart. Their strengths are annular size compatibility and large coaptation gaps, low annular interaction, and possibly shorter procedural times with less sophisticated imaging.

### CroiValve DUO

The DUO Transcatheter Tricuspid Coaptation Valve System (CroiValve Ltd.) is a novel device designed to treat TR by enhancing native leaflet coaptation without annular fixation. It comprises a porcine pericardial coaptation valve, an adjustable catheter system, and a self-expanding stent anchored in the SVC.^59^ The valve is suspended between the native leaflets, which coapt against an outer skirt to reduce regurgitation, while a central valve maintains diastolic flow. This anchoring approach avoids interference with right heart structures or the conduction system and allows flexible positioning tailored to patient anatomy. The system is delivered via a 22Fr sheath through the right internal jugular vein. The TANDEM II EFS (NCT05913908) is currently ongoing and aims to enrol 15 patients to evaluate freedom from device- or procedure-related MAEs.

### Tripair

The Tripair device (Coramaze Technologies, Tikva, Israel) features a central spacer supported by a flexible central column, anchored by an atraumatic crown that rests in the right atrium. It is delivered transfemorally, allows for rapid deployment without the need for complex imaging, and is fully retrievable.^60^ The first-in-human experience with the Tripair Enhanced System was performed in May 2025, yielding encouraging results.^61^ The pivotal study is expected to start in 2027 and conclude in 2029.

## Transcatheter tricuspid annuloplasty

Transcatheter tricuspid annuloplasty is based on the most adopted surgical tricuspid repair approach. Implantation of a tricuspid ring either surgically or percutaneously would seem to be the most physiologic way to repair a valve in which annular dilatation is one of the main mechanisms of dysfunction. Transcatheter tricuspid devices reduce annular diameters through suture or anchor systems that are mainly positioned in the posterior and anterior aspects of the tricuspid annulus. Nonetheless, transcatheter tricuspid annuloplasty systems have not been widely accepted as a first-line option for percutaneous TR treatment due to either the lack of relevant TR reduction or the technical procedural complexity. Over the last years, different systems have been developed and tested in several trials. Among them, the Cardioband Tricuspid Repair System is the only technique with certain penetration in some high-volume and experienced European centers.

### The cardioband tricuspid repair system

The Cardioband annuloplasty system (Edwards Lifesciences, Irvine, CA, US) was the only transcatheter tricuspid valve annuloplasty device approved in Europe (CE mark 2018) up to April 2025.^62^ The original system has been abandoned as a second generation named Cardioband FIT Valve Repair System is currently being tested in a multi-centre, prospective and non-randomized study designed to evaluate its performance and safety (NCT03382457).

**Figure 3:**
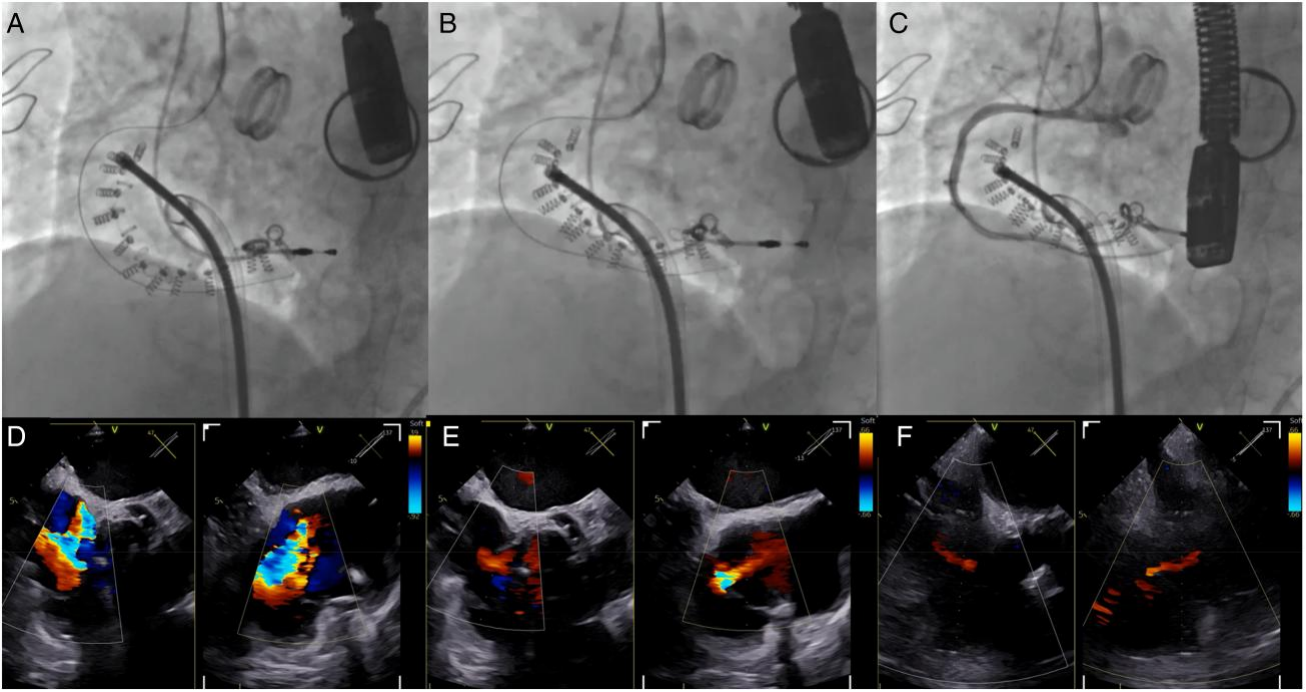
Annuloplasty with the cardioband system. Progressive cinching of the tricuspid annulus with the Cardioband system on angiographic views (A–C), with subsequent reduction in tricuspid regurgitation on echocardiographic images (D–F).

The Cardioband system is a 28Fr *trans*-femoral device. A tricuspid band (four sizes fitting the annulus diameter) is anchored towards the lateral side of the annulus through dedicated screws (14 to 17 screws), which are implanted individually and sequentially (*Figure 3*). After ensuring band anchoring, a progressive cinching provides modulable reduction of the tricuspid annular diameter. The procedure requires screening with both transoesophageal echocardiography and CT-scan.

The Tri-REPAIR trial explored the feasibility and safety of the original system^25^ while the Tri-BAND trial tested the procedural success.^63^ Although no mortality was reported and procedural success was close to 100%, around 20% of patients experienced some MAE. Since then, several registries have been published with better outcomes,^64,65^ and the commercial use of the Cardioband system has been reserved for high-volume and experienced centres with a specific interest in tricuspid repair beyond the T-TEER systems.

The potential advantages of the system are the capacity to provide a repair option in valves in which T-TEER is not optimal (challenging leaflet anatomy or large gaps) and the absence of future interference with other transcatheter tricuspid systems, such as T-TEER or orthotopic/heterotopic TTVR. In addition, the combination of an initial tricuspid annuloplasty to reduce the coaptation gap and subsequently undergo T-TEER might be a valid alternative to offer a repair option in valves with large coaptation gaps.^66^ In contrast, the main drawbacks of the procedure are the relatively high rate of rejection (around 30%) for excessive annular dilatation or the potential risk of right coronary artery perforation. Another potential limitation relies on the imaging requirements as high-quality transoesophageal echocardiography imaging is needed to ensure individual screw anchoring. Image quality is, however, only required in the atrial side of the valve (no need for leaflet imaging) and intracardiac echocardiography is very useful in difficult cases.^67^

### Other transcatheter tricuspid annuloplasty systems or techniques

- *The TriCinch* system is one of the first percutaneous annuloplasty systems that was abandoned due to the high rate of MAEs. The system generated tension over a tricuspid anchor through a connected stent that was implanted in the inferior vena cava.
- *The Trialign* system was also one of the first percutaneous annuloplasty systems. It was a transjugular suture-based tricuspid valve annuloplasty system that reduced tricuspid annular diameter through tissue plication.
- *The Da Vingi TR system* is a two-step 22Fr *trans*-jugular system in which a tricuspid ring is implanted through several anchors and posteriorly cinched.
- *The Minimally Invasive Annuloplasty (MIA)* employs multiple polymeric hooks with thermoplastic polymer that generate compression to the annulus.
- *The K-Clip* is a *trans*-jugular system that uses one or more clips that are positioned over the tricuspid annulus in order to reduce its diameter and limit the progressive dilatation.

## Conclusions

TR has transitioned from an afterthought to a key emphasis of structural heart therapy. Concurrently, the transcatheter toolkit has evolved from annular and edge-to-edge repair to full valve replacement and now has choices within a wider range of anatomies. New trial data, registries, and real-world programmes consistently demonstrate substantive TR reduction, symptomatic and quality of life improvement, and steadily enhancing safety profiles. Anatomic variability and patient selection and timing are critical challenges remaining, long-term durability and haemodynamic effects on the right ventricle and pulmonary circuits and necessary head-to-head comparisons within platforms. Despite these challenges remaining to be addressed, the present and immediate future are strong and bright with the latest-generation devices and refined selection criteria and robust evidence on hard outcomes on the cusp of truly individualized therapy by heart teams.

## Data Availability

The data underlying this article will be shared on reasonable request to the corresponding author.
